# The Correlation between Using Social Networks and the General Health of Multiple Sclerosis Patients

**DOI:** 10.1155/2020/2791317

**Published:** 2020-07-09

**Authors:** Atefeh Basirat, Hadi Raeisi Shahraki, Hamid reza Farpour, Leila Habibi

**Affiliations:** ^1^Department of Biostatistics, Faculty of Medicine, Shiraz University of Medical Sciences, Shiraz, Iran; ^2^Department of Epidemiology and Biostatistics, Faculty of Health, Shahrekord University of Medical Sciences, Shahrekord, Iran; ^3^Bone and Joint Diseases Research Center, Department of Physical Medicine and Rehabilitation, Shiraz University of Medical Sciences, Shiraz, Iran; ^4^Shiraz Geriatric Research Center, Shiraz University of Medical Sciences, Shiraz, Iran; ^5^PhD of media management, University of applied sciences and technology, Iran; ^6^Department of media Management, Faculty of management, University of Tehran, Tehran, Iran

## Abstract

**Background:**

Multiple sclerosis (MS) threatens the patients' independency and ability to effectively participate in the society. The aim of this study was to determine the correlation between using social networks on the general health of multiple sclerosis patients.

**Methods:**

This study was performed on 80 MS patients referring to Shiraz University of Medical Sciences, Chamran, and Imam Reza Hospital in 2017, whose condition had improved and were treated by a specialist physician. Tools for data collection were general health questionnaire (GHQ-28) and social networks use questionnaires.

**Results:**

Amongst the 80 individuals with MS, 65 (81.3%) were female and 15 (18.8%) were male. Our results suggest that patients with higher levels of education had higher levels of health (*P* = 0.01). Telegram and WhatsApp, respectively, had a positive correlation with general health (*P* = 0.007, *P* = 0.007), anxiety (*P* = 0.003, *P* = 0.028), and social dysfunction (*P* = 0.007, *P* = 0.007). WhatsApp with 33.64% was the most popular application among MS patients. The correlation between general health and duration of using social networks was not statistically significant (*r* = 0.22, *P* = 0.06). Also, no significant correlation was found between the duration of using social networks and the general health (*P* = 0.62).

**Conclusion:**

Our findings suggest that social networks, especially Telegram and WhatsApp, had a positive correlation with general health, anxiety, and social dysfunction of patients. Therefore, the use of social networks can be considered as a suitable option in reducing the aforementioned concerns among patients with MS. On the other hand, general health and mood status might as well influence the use of social network in MS patients.

## 1. Introduction

Multiple sclerosis (MS) is the most common immune-mediated disorder affecting the central nervous system [1]. It threatens the patients' independency and ability to effectively participate in the society [2]. Supporting social ties and relationships between MS patients and others is crucial [3]. Several studies have shown that social support is an important source for patients with MS [4]. Social support is shown to be positively associated with physical and mental health status in patients with MS [5]. Social support is a mutual process characterized by a network of interpersonal relationships [6]. Considering the movement restrictions related to MS [7] and patients' limited ability to participate in support communities in an actual space, virtual communities might be a potential route for exchanging social support [8]. Nowadays, the positive effect of psychological interventions on improving chronic diseases has been confirmed [9, 10]. In a research, 2007 on hope in advanced diseases, Mum Hong showed that support from family and friends, religion, acceptance, self-awareness, and knowledge on the current conditions are important factors for increasing hope and fighting the challenges [11]. Compared to usual care, the internet, as a source, presents potentially better answers to the patients' needs for independency, experience, and communication [12]. The positive effects of media functioning in the field of health have been proven in many studies [13–15]. Hope is one of the main resources for the adaptation capability of the chronic patients to survive, which has impacts on the individual's personal attitudes, his/her health status [16]. These patients are usually confronted with a vast array of such negative feelings, like anxiety, anger, and depression, especially when the disease causes the patients' roles and their activities to decline and their social relations to change [17]. Studies have indicated that approximately 25 to 40 percent of the patients with MS suffer from anxiety [18], of which life expectancy and revitalization can enhance coping with the disease [19]. Just as the social networks can put people under severe psychological pressure, it also has the ability to relieve their stress, bring about peace, and reduce their depression [20].

Diminished subjective well-being was the most common effect found in patients, using social networks for health-related reasons. Examples include “demoralization” [21], “hurt feelings due to negative feedback” [22], and “increased feelings of anxiety” [23]. Loss of privacy was also mentioned in only one article [22]. Addiction to social networks was identified in one article [23]. Therefore, social networks can have positive or negative effects on MS patients.

At this time, considering the known functions of social networks and their interactive nature, we might be able to use them in order to satisfy the needs of MS patients. Culture and structure of different societies are the two factors strongly influencing the components of the questionnaire based on several studies as in the study of Taghavi, using public health on Shiraz University students and derived the four factors of depression, anxiety, social dysfunction, and physical symptoms with the Cronbach alpha of 0.90. Also, 50.8% of the total variance was explained by these four components [24]. Similarly, Molavi et al. in a study among students in Isfahan University extracted the four components, and the reliability of the questionnaire was 0.91 [25]. In another study on patients with psychiatric disorders by Ebrahimi et al., four factors were extracted, and the Cronbach alpha was 0.97 [26]. In the study carried out by Sadjjadi et al., the factor structure of the GHQ in patients with a traumatic brain injury was studied, and the reliability coefficient was 0.89. The four extracted factors were anxiety and insomnia, social dysfunction, depression, and physical symptoms [27]. Also, Ghanbarnejad et al. evaluated the reliability and validity of GHQ-28 by examining four different methods [28] on dermatologic patients. Moreover, in assessing the students in East Azarbaijan by standardizing GHQ-28 questionnaire, Javanmard et al. found four components from factor structure including physical symptoms, anxiety and sleep disorders, social dysfunction, and depression, respectively, by the reported reliability coefficient of 0.87 [29]. Therefore, in this research, we seek to investigate the correlation between social networks and general health of svMS patients.

## 2. Methods

This is a cross-sectional descriptive-correlational study performed on patients with MS admitted to Shiraz University of Medical Sciences affiliated hospitals of Chamran and Imam Reza in 2017, whose condition had improved and were treated by a specialist physician. Given the main aim of this research and considering *r* = 0.31 (correlation between the time of using social networks and general health), type one and two error 0.05 and 0.20, respectively, the sample size was calculated at 80, using the following formula:

(1) $$\begin{array}{c} N={\left[\left(Z_{\alpha }+Z_{\beta }\right)/C\right]}^{2}+3\;\text{where }C=0.5\;\ln\;\left[\left(1+r\right)/\left(1-r\right)\right] \end{array}$$

Patients with MS admitted to Chamran and Imam Reza Hospitals were selected through convenient sampling. Patients diagnosed with MS who had the ability to use social networks were included in this study.

Inclusion criteria are as follows: MS patients with the ability to use the Internet and use at least one social networks (Facebook, Twitter, blogs, forums, and text messages, Telegrams, WhatsApp, YouTube, Club, Instagram, and Viber) and ready to participate in the interview. Exclusion criteria were the inability to use the Internet and unwillingness to cooperate. Patients with simultaneous damage to the nervous system due to rheumatologic diseases, such as lupus, stroke, and traumatic brain injury were also excluded. At first, 112 patients were selected of which 14 did not use any of the mentioned mobile applications. Then, 17 were not willing to complete the questionnaire, and 1 had a traumatic history; consequently, 80 patients were entered in the study.

Data collection instruments consisted of general health questionnaire (GHQ) and social networks use questionnaire. Moreover, demographic characteristics such as age, gender, education, marital status, occupation, the involved body part, and duration of illness were questioned. Goldberg and Hiller's general health questionnaire was designed in 1979. This questionnaire has been translated into different languages, and its reliability, validity, and factor structure has been checked in different cultures. The 28-item version of the GHQ is the only version that provides subscale measures of more specific domains of psychopathology (Goldberg & Hillier, 1979). Social networks questionnaire was developed by Goldberg in 1978 and has been translated into 38 languages. This questionnaire, GHQ-28, consisting 28 items, was developed as a screening tool for the ones who are likely to have or are at risk of developing psychiatric disorders. It measures emotional distress in medical settings. This questionnaire is divided into four subscales such as somatic symptoms (items 1–7), anxiety/insomnia (items 8–14), social dysfunction (items 15–21), and severe depression (items 22–28), through factor analysis. The items are scored through Likert scale (1–4, and), and consequently, the total score of a person varies from 28 to 112. In both scoring methods, higher scores indicate better mental health. After receiving verbal consent from the patients, the questionnaire was completed. Social networks use questionnaire includes Facebook, Twitter, blogs, forums, text messages, telegram, WhatsApp, YouTube, clubs, Facebook, Instagram, and Viber. Descriptive statistics methods, such as frequency, percentage, mean, and standard deviation were used to describe the data. Spearman's correlation test was used to determine the correlation between the use of social networks and general health of MS patients. Comparisons between different groups of patients were done, using ANOVA test or independent *t*-test for the continuous variables, and by Kruskal-Wallis or Mann–Whitney for the ordinal variables. Chi-square or Fisher's exact test was used to investigate the associations among categorical variables. Data analysis was performed, using SPSS 16.0, and *P* < 0.05 was considered to be statistically significant.

## 3. Results

**Figure 1:**
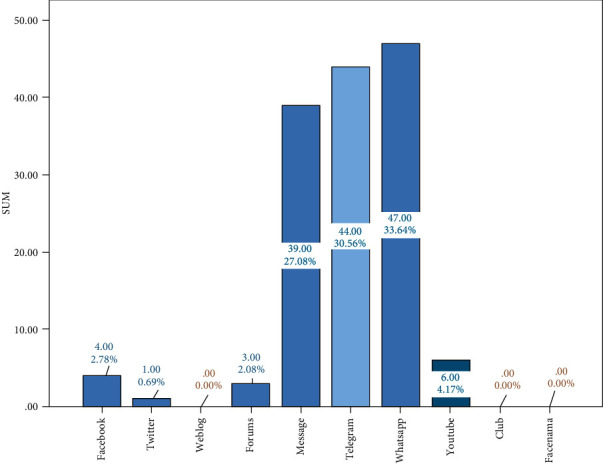
The percentage of MS patient using each social networks.

**Figure 2:**
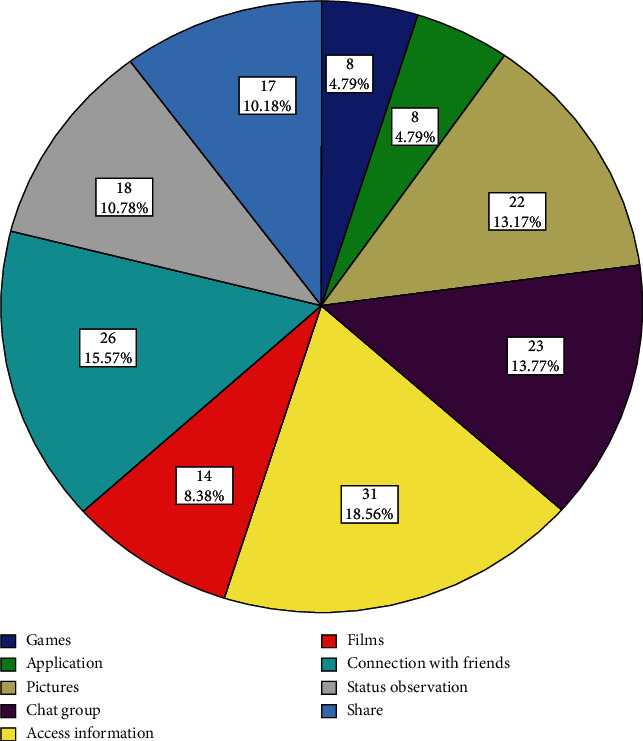
The goal of using social networks.

In this research, 80 patients with MS were studied amongst whom 65 (81.3%) were female and 15 (18.8%) were male. The patients' mean age was 38.0 ± 10.4 years, and the mean duration of their weekly use of social networks was 1.7 ± 2.1 hours; most of them were married (78.8%) and had diplomas (37.5%). All demographic characteristics of the MS patients are presented in Table 1. As shown in Figure 1, WhatsApp with a proportion of 0.33 was the most popular application among the MS patients, and using social networks has been most appealing for patients to have quick access to information (18.56%), to communicate with friends (15.57%), chat groups (13.77%), and pictures (13.17%) (Figure 2). It should be noted that the range of time for social networks use was from 0 to 10 hours in a week.

Independent *t*-test showed that there was no significant difference between the males and females (*P* = 0.86), single and married patients (*P* = 0.61), patients in different age group (*P* = 0.96), and duration of disease (*P* = 0.43) in terms of general health. Also, there was a significant association between educational level and social networks usage (*P* < 0.001). Our results suggest that patients with higher levels of education had a higher level of health (*P* = 0.01, Table 1).

Table 2 shows the result of comparing the general health and its dimensions between users and nonusers of different applications. Telegram, WhatsApp, and YouTube users had higher scores. The aforementioned differences were significant for general health and anxiety and social dysfunction dimensions.

Spearman's correlation between the mean of hours using social networks and general health was 0.217, which was slightly higher than 0.05. Moreover, no significant correlation was detected between the mean of hours using social networks and dimensions of general health. Also, the result of the ANOVA test in Table 3 shows that there was no significant difference between the general health of patients and its various dimensions in the three groups (the time scale of using social networks in terms of hours per day). This means that the duration of using social networks does not affect the general health of MS patients.

## 4. Discussion

The main aim of this study was to investigate the correlation between using social networks and the general health of MS patients. A number of researches suggest that social factors play an important role in maintaining health and adjusting to a range of clinical conditions. Therefore, social networks may affect the health of these patients.

In this study, the results showed that among subgroups of demographic characteristics of the MS patient including gender, marital status, age, duration of infection, and education, merely the level of education had a significant correlation with the general health status, especially in the area of depression and social dysfunction. Those who had a bachelor's degree, MS, and PhD had better social functioning and less depression compared to the other two groups. Some evidence is indicative of the fact that social networks can have a positive influence on health and improve health outcomes for people with depression [20]. Other studies have verified the effect of education on depression [29]. On the other hand, according to the results of a study [30], there was a significant relationship between MS and social problems. The results of the research by Osborne et al., Patten et al. [31, 32], Zabad, and Cation, also confirmed the meaningful relationship. Research conducted by Janner et al. 2004 showed that MS patients experienced a high level of depression and had low general health [33, 34]. This may be due to the fact that MS is a chronic disease like cancer. In an article on the effect of the general health on cancer patients, cancer had the most negative impact in general, especially in the area of social dysfunction and anxiety [35]. In the present study, the general health of MS patients was not significant in terms of gender, age, and marital status. In a study [36], the authors showed no significant difference between the general health of MS patients in male and female participant. In reference books, the patients' age ranged between 20 and 40, [37] is consistent with the results of this study.

Although in the current study, the correlation between the duration of using social networks (in hour) and general health dimensions was not statistically significant, this might be due to the small sample size. Most of the patients in this study used social networks including text message, Telegram, WhatsApp, and Facebook; in addition, the results showed that the general health of people using the telegram and WhatsApp was significantly different from those who did not use them. They also had a better social performance and less anxiety and insomnia.

The results of a previous study showed that the patients using social networks for support enhanced their psychological well-being [38]. Social networks can provide psychosocial support and the ability to communicate with similar individuals with MS to reduce stress and increase life expectancy in people with this disease. In this study, the main purpose of patients for using social networks was for entertainment and staying in touch with friends and family members, and their least important aim was to find and communicate with new people. However, [13] the main purpose of patients was to use Instagram for gathering information from people whose relatives have cancer. The main source for the majority of patients was the use of mobile social networks and the minority of them used laptops, apparently because the mobiles are more accessible than computers and laptops.

The most appealing motivation for using social networks was to have quick access to information to communicate with friends, follow discussion groups, and view the status of others. The reasons for those who did not use social networks were lack of awareness on how to use the media or their lack of accessibility. They also mentioned that they were not very sociable. Given the great potential of social networks, health policy makers should provide patients with correct scientific information through introducing and managing useful websites and channels to help the patients not to endanger their health by getting in touch with unauthorized websites and fraudulent ads. Also, the proper use of social networks and scientific interactions between the patient and the medical staff can prevent their direct referral for simple ill-considered issues, which saves time and energy and reduces the costs for transportation and caregivers.

In this study, individuals were more likely to use WhatsApp, Telegram, and Message, perhaps because access to these media is easier for the general public. Since the research sample was from the patients referring to Shiraz University of Medical Sciences affiliated hospitals of Chamran and Imam Reza in 2017, and there was no access to all the MS patients in Shiraz, generalizing the findings should be done with caution. Hence, it is suggested that this research to be conducted in a wider range and larger sample size.

There was a positive correlation between the score of general health, depression symptoms, and social dysfunction of patients with MS with the duration of using social networks. Social networks, especially Telegram and WhatsApp, had a positive correlation with general health, anxiety, and social dysfunction of multiple sclerosis patients. Although there is no proven “treatment effect,” the current study found that there is a positive association between using social networks and public health of patients.

## Figures and Tables

**Table 1:** Comparison of general health and its dimensions among subgroups of demographic characteristics of the MS patient.

Variable	Sub group	Number (%)	General health	Depression	Anxiety and insomnia	Somatization	Social dysfunction
Sex	Male	15 (18.8)	79.27 ± 14.44	18.47 ± 4.12	19.6 ± 3.99	19.067 ± 4.11	22.13 ± 6.2
	Female	65 (81.2)	80.00 ± 14.43	17.53 ± 5.01	19.31 ± 5.13	19.02 ± 3.47	24.15 ± 4.79
*P* value			0.86	0.50	0.84	0.96	0.17
Marital status	Single	17 (21.3)	81.5 ± 12.65	18.87 ± 4.11	19.94 ± 3.64	19.187 ± 3.98	23.50 ± 5.57
	Married	63 (78.8)	79.42 ± 14.82	17.40 ± 4.99	19.22 ± 5.20	18.98 ± 3.49	23.82 ± 5.037
*P* value			0.61	0.28	0.61	0.84	0.83
Age	<30	21 (26.3)	80.6 ± 13.35	18.95 ± 4.58	18.85 ± 4.18	19 ± 3.98	23.8019 ± 5.27
	30-50	51 (63.8)	79.54 ± 15.20	17.35 ± 4.89	19.73 ± 4.80	19.04 ± 3.64	23.42 ± 5.28
	>50	8 (10.0)	79.87 ± 12.85	16.75 ± 5.17	18.50 ± 7.25	19 ± 2.27	25.62 ± 3.66
*P* value			0.96	0.39	0.70	0.999	0.53
Duration of disease	<1 year	15 (18.8)	78.6 ± 16.68	18.93 ± 5.12	18.80 ± 5.38	18.27 ± 4.26	22.60 ± 5.22
	2-10 years	35 (43.8)	77.90 ± 15.08	16.61 ± 4.55	18.77 ± 4.82	18.96 ± 3.67	23.55 ± 5.45
	>10 years	30 (37.5)	82.50 ± 12.25	18.23 ± 4.91	20.27 ± 4.76	19.47 ± 3.14	24.53 ± 4.74
*P* value			0.43	0.24	0.44	0.57	0.48
Education	Undergraduate	30 (37.5)	76.07 ± 15.24	16.80 ± 5.15	18.33 ± 5.52	18.80 ± 3.25	22.13 ± 5.71
	Diploma	26 (32.5)	77.84 ± 13.23	16.84 ± 3.51	18.88 ± 4.59	18.32 ± 4.31	23.80 ± 5.39
	MS or Ph. D	24 (30.0)	87.67 ± 11.54	20.05 ± 5.15	21.43 ± 3.77	20.19 ± 2.87	26 ± 2.60
*P* value			0.01	0.03	0.07	0.19	0.03

**Table 2:** Comparison of general health and its dimensions between users and nonusers of different applications.

Social network	Use social network	Number (%)	General health	Depression	Anxiety and insomnia	Somatization	Social dysfunction
WhatsApp	Yes	47 (58.8)	83.67 ± 12.94	18.44 ± 4.80	20.44 ± 4.59	19.60 ± 3.58	25.19 ± 3.92
	No	33 (41.3)	74.88 ± 14.73	16.76 ± 4.79	17.97 ± 5	18.27 ± 3.48	21.88 ± 5.90
*P* value			0.007	0.133	0.028	0.108	0.007
Telegram	Yes	44 (55)	84.02 ± 13.27	18.60 ± 14.74	20.90 ± 4.30	19.38 ± 3.83	25.19 ± 3.92
	No	36 (45)	75.22 ± 14.22	16.72 ± 4.82	17.67 ± 5.02	18.64 ± 3.29	21.88 ± 5.90
*P* value			0.007	0.091	0.003	0.374	0.007
Message (Tel)	Yes	39 (48.8)	81.43 ± 13.56	17.59 ± 4.77	19.70 ± 4.22	19.65 ± 3.75	24.49 ± 4.63
	No	41 (51.3)	78.36 ± 15.06	17.82 ± 4.96	19.05 ± 5.51	18.44 ± 3.35	23.05 ± 5.51
*P* value			0.354	0.556	0.556	0.141	0.224
YouTube	Yes	6 (7.5)	82 ± 14.35	17.75 ± 4.5	17.75 ± 4.79	21.25 ± 2.75	25.25 ± 4.86
	No	74 (95.5)	79.74 ± 14.43	17.71 ± 4.89	19.46 ± 4.9	18.90 ± 3.59	23.67 ± 5.15
*P* value			0.727	0.133	0.028	0.108	0.005
Facebook	Yes	4 (5)	82.00 ± 14.35	17.75 ± 4.5	19.46 ± 4.92	18.90 ± 3.59	23.67 ± 5.15
	No	76 (95)	79.74 ± 14.43	17.71 ± 4.88	17.75 ± 4.79	21.25 ± 2.75	25.25 ± 4.86
*P* value			0.761	0.987	0.501	0.202	0.551

**Table 3:** Descriptive statistics of general health and its dimensions separated by different durations of using social networks (in hour).

Group	*N* (%)	General health	Depression	Anxiety and insomnia	Somatization	Social dysfunction
<1(hour)	19 (0.37)	83.26 ± 13.55	19.05 ± 4.48	20.53 ± 4.22	19.53 ± 3.50	24.16 ± 4.75
1-6(hours)	29 (0.57)	83 ± 13.48	18.34 ± 4.96	20.17 ± 4.79	19.21 ± 3.85	25.27 ± 4.25
7-10(hours)	3 (0.06)	80 ± 8.71	16 ± 2	20 ± 1	21 ± 3	23 ± 4
*P* value		0.62	0.179	0.131	0.449	0.085

## Data Availability

We can send our dataset for the journal without any limitation.
